# Cardiovascular magnetic resonance‐assessed fast global longitudinal strain parameters add diagnostic and prognostic insights in right ventricular volume and pressure loading disease conditions

**DOI:** 10.1186/s12968-021-00724-5

**Published:** 2021-04-01

**Authors:** Shuang Leng, Ru-San Tan, Jiajun Guo, Ping Chai, Gangcheng Zhang, Lynette Teo, Wen Ruan, Tee Joo Yeo, Xiaodan Zhao, John C. Allen, Ju Le Tan, James W. Yip, Yucheng Chen, Liang Zhong

**Affiliations:** 1 National Heart Research Institute Singapore, National Heart Centre Singapore, 5 Hospital Drive, 169609 Singapore, Singapore; 2 Duke-NUS Medical School, National University of Singapore, Singapore, Singapore; 3 Cardiology Division, Department of Medicine, West China Hospital, Sichuan University, No. 37 Guoxue Alley, Wuhou District, Chengdu City, 610041 Sichuan People’s Republic of China; 4 Department of Cardiology, National University Heart Centre Singapore, Singapore, Singapore; 5 Yong Loo Lin School of Medicine, National University of Singapore, Singapore, Singapore; 6 Wuhan Asia Heart Hospital, Wuhan City, Hubei People’s Republic of China; 7 Department of Diagnostic Imaging, National University Hospital, Singapore, Singapore

**Keywords:** Cardiovascular magnetic resonance, Right ventricular function, Strain, Volume overload, Pressure overload

## Abstract

**Background:**

Parameters of myocardial deformation may provide improved insights into right ventricular (RV) dysfunction. We quantified RV longitudinal myocardial function using a fast, semi-automated method and investigated its diagnostic and prognostic values in patients with repaired tetralogy of Fallot (rTOF) and pulmonary arterial hypertension (PAH), who respectively exemplify patients with RV volume and pressure overload conditions.

**Methods:**

The study enrolled 150 patients (rTOF, n = 75; PAH, n = 75) and 75 healthy controls. RV parameters of interest were fast global longitudinal strain (GLS) and strain rates during systole (GLSR_s_), early diastole (GLSR_e_) and late diastole (GLSR_a_), obtained by tracking the distance from the medial and lateral tricuspid valve insertions to the RV epicardial apex on cine cardiovascular magnetic resonance (CMR).

**Results:**

The RV fast GLS exhibited good agreement with strain values obtained by conventional feature tracking approach (bias − 4.9%, error limits (± 2·standard deviation) ± 4.3%) with fast GLS achieving greater reproducibility and requiring reduced analysis time. Mean RV fast GLS was reduced in PAH and rTOF groups compared to healthy controls (PAH < rTOF < healthy controls: 15.1 ± 4.9 < 19.3 ± 2.4 < 24.4 ± 3.0%, all *P* < 0.001 in pairwise comparisons). In rTOF patients, RV fast GLS was significantly associated with metabolic equivalents, peak oxygen consumption (PVO_2_) and percentage of predicted PVO_2_ achieved during cardiopulmonary exercise testing. Lower RV fast GLS was associated with subnormal exercise capacity in rTOF (area under the curve (AUC) = 0.822, sensitivity = 72%, specificity = 91%, cut-off = 19.3%). In PAH patients, reduced RV fast GLS was associated with RV decompensated hemodynamics (AUC = 0.717, sensitivity = 75%, specificity = 58%, cut-off = 14.6%) and higher risk of clinical worsening (AUC = 0.808, sensitivity = 79%, specificity = 70 %, cut-off = 16.0%).

**Conclusions:**

Quantitative RV fast strain and strain rate parameters assessed from CMR identify abnormalities of RV function in rTOF and PAH and are predictive of exercise capacity, RV decompensation and clinical risks in these patients.

*Trial registry* Clinicaltrials.gov: NCT03217240

**Supplementary information:**

The online version contains supplementary material available at 10.1186/s12968-021-00724-5.

## Introduction

Analysis of right ventricular (RV) performance has not been studied as exhaustively as that of the left ventricle (LV). An important cause of RV dysfunction is chronic pressure overload induced by conditions like pulmonary arterial hypertension (PAH). On the other hand, RV dysfunction from chronic volume overload is a complication in residual pulmonary valve regurgitation after tetralogy of Fallot repair (rTOF) [1].

Compared with the LV, quantitative assessment of RV function is challenging due to the latter’s more complex anatomy. Cardiovascular magnetic resonance (CMR) is the reference standard for non-invasive assessment of RV structure and function [2]. It is able to capture the complex 3-dimensional (3D) anatomy without geometric assumption. Further, CMR allows for measurement of deformation in 3D using cine CMR feature tracking (FT) [3]. Prior study demonstrated that quantification of myocardial deformation by FT detects pulmonary hypertension-induced RV dysfunction and confers independent prognostic value after adjustment for other risk factors including RV ejection fraction (RVEF) [4]. However, in subjects with relatively vigorous tricuspid annular motion, contour tracking of the RV free wall segment adjacent to the tricuspid valve in long-axis view is degraded and becomes a source of error for the measurement of longitudinal strain [5]. Notably, the existence of different FT analysis algorithms by various vendors exacerbates measurement variability [6], and underscores the need for a harmonized vendor-agnostic approach to CMR FT.

We have developed a fast, semi-automated approach for quantifying longitudinal strain and strain rate (SR) from standard cine CMR images and demonstrated its effectiveness in assessing longitudinal function of left atrium [7, 8], right atrium (RA) [9], and LV [10]. The current study aimed to apply this approach in RV and to investigate the diagnostic and prognostic values of RV longitudinal strain measurements in rTOF and PAH patients, who respectively exemplify patients with RV volume and pressure overload conditions compared with healthy controls.

## Methods

### Study population

The study prospectively enrolled 75 patients with rTOF, 75 patients with PAH, and 75 healthy controls to undergo CMR. Additionally, all rTOF patients underwent same-day cardiopulmonary exercise testing (CPET). Exclusion criteria for rTOF included presence of intracardiac shunt, pulmonary stenosis with peak pressure gradient > 30 mmHg, supraventricular arrhythmia, significant ventricular arrhythmia, and prior pulmonary valve replacement. The diagnosis criteria for PAH was based on right heart catheterization [11]: (1) mean pulmonary artery pressure (mPAP) > 25 mmHg; (2) pulmonary capillary wedge pressure ≤ 15 mmHg; and (3) pulmonary vascular resistance (PVR) > 3 Wood units. PAH patients were stratified into two sub-groups according to invasively measured mean RA pressure [12]: PAH with hemodynamically compensated RV function (PAH-C) defined by mean RA  pressure < 10 mmHg; and PAH with hemodynamically decompensated RV function (PAH-D) defined by mean RA pressure ≥ 10 mmHg. In addition, PAH patients were classified as low, intermediate or high risk of clinical worsening in accordance with guideline-recommended parameters [11]: World Health Organization functional class, six-minute walking distance (6MWD), N-terminal pro-brain natriuretic peptide, RA area, RA pressure, and cardiac index. The protocol had been approved by the SingHealth Centralised Institutional Review Board and the Institutional Review Boards of West China Medical Centre of Sichuan Hospital and Wuhan Asia Heart Hospital. Informed consent was obtained from all participants.

### CMR acquisition and volumetric analysis

Enrolled subjects underwent CMR on a 3 T CMR scanner (Ingenia, Philips Healthcare, The Netherlands or MAGNETOM Tim Trio, Siemens Healthineers, Erlangen, Germany), or 1.5 T CMR scanner (Avanto, Siemens Healthineers). End-expiratory breath hold balanced steady-state free precession cine images were acquired in short- and long-axis LV views. The standard 4-chamber view was prescribed from the vertical long-axis through the apex and centre of the mitral and tricuspid valves. Typical parameters for the 3T Philips scanner were as follows: repetition time (TR)/echo time (TE), 3/1 ms; matrix, 240 × 240; flip angle, 45°; field of view, 300 × 300 mm; pixel bandwidth, 1776 Hz; pixel spacing, 1.25 × 1.25 mm; slice thickness, 8 mm; number of frames, 30/40 per cardiac cycle; actual temporal resolution (TR × turbo factor), 42 ms. Parameters for the 3T Siemens scanner were: TR/TE, 3.4/1.3 ms; matrix, 162 × 192; flip angle, 50°; field of view, 270 × 320 mm; pixel bandwidth, 1532 Hz; pixel spacing, 1.67 × 1.67 mm; slice thickness, 8 mm; number of frames, 25 per cardiac cycle; actual temporal resolution, 40.6 ms. Parameters for the 1.5T Siemens scanner were: TR/TE, 3.3/1.1 ms; matrix, 156 × 192; flip angle, 80°; field of view, 276 × 340 mm; pixel bandwidth, 930 Hz; pixel spacing, 1.77 × 1.77 mm; slice thickness, 6 mm; number of frames, 25 per cardiac cycle; actual temporal resolution, 39.8 ms.

Volumetric analysis was performed by the cardiologists in respective centres blinded to other CMR measurements. Endocardial surfaces were manually traced from the stack of short-axis cine images to obtain ventricular end-diastolic volume (EDV), end-systolic volume (ESV), and to calculate stroke volume (SV) and ejection fraction (EF). Papillary muscles and trabeculae were included in the blood volume [13].

### RV fast global longitudinal strain

**Fig. 1:**
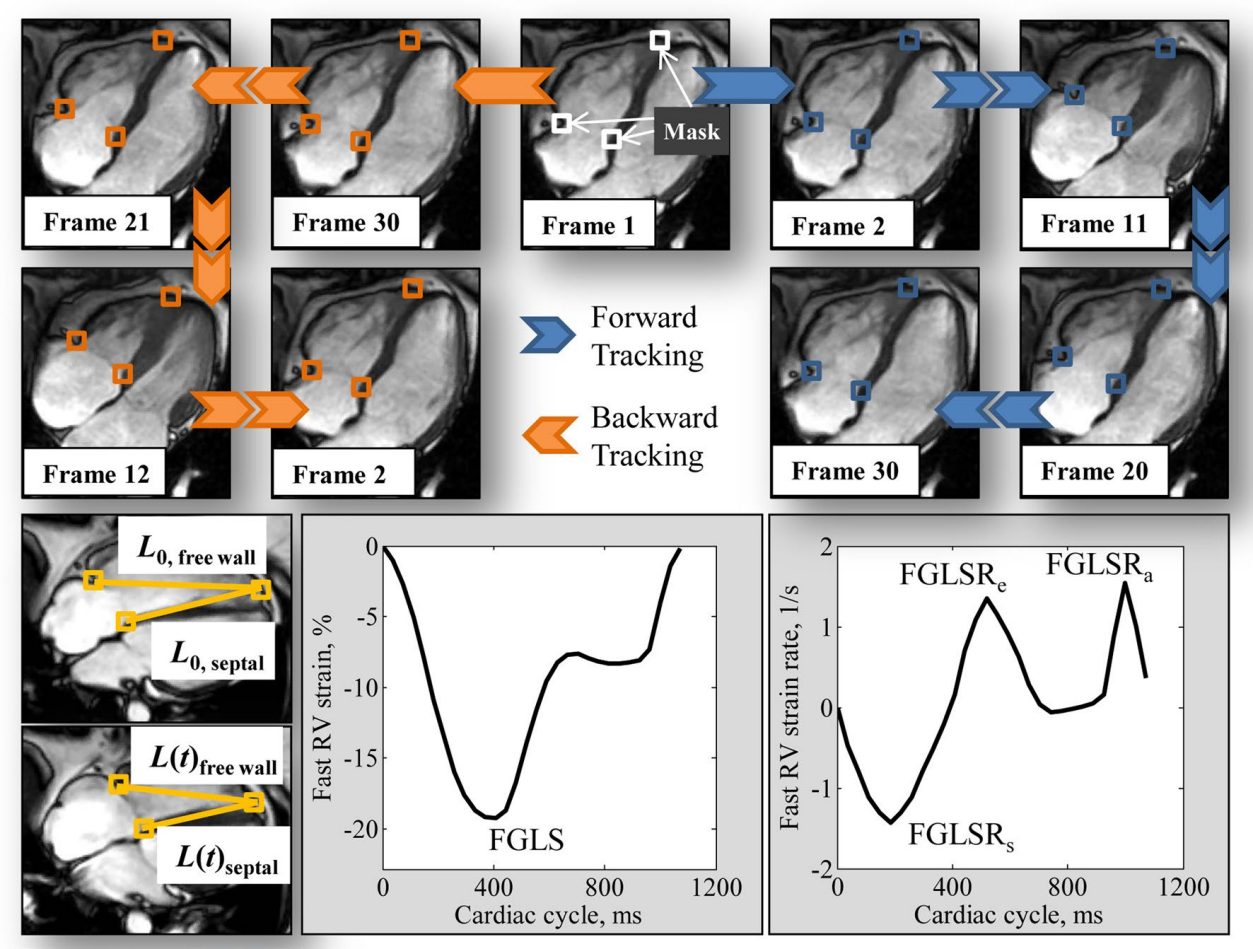
*Top* Semi-automatic tracking of tricuspid annular insertions and right ventricular (RV) epicardial apex, *Bottom* The strain of each wall (RV septal and free wall) was calculated using the presented strain formula. RV fast global longitudinal strain (FGLS) and peak strain rates (FGLSR_s_, FGLSR_e_, and FGLSR_a_) were extracted from the strain and strain rate curves

Fast RV strain assessment was performed on CMR 4-chamber view by one reader (S.L.) blinded to clinical characteristics of participants and other CMR measurements. The distance (*L*) from the medial and lateral tricuspid valve insertions to the RV epicardial apex was automatically tracked throughout the cardiac cycle (Fig. 1). Briefly, squares (called masks, Fig. 1) containing the anatomical points of interest (tricuspid valve insertions and RV epicardial apex) were manually drawn in the RV end-diastolic frame (1st frame). These masks were then automatically tracked forward in time, then backward [14, 15]. The final trajectory result was calculated by averaging those from both forward and backward tracking. More details about the anatomical point tracking and the methodological considerations and robustness analysis on RV apical point selection are available in Additional file 1.

The fast RV strain assessment approach incorporates the time-varying distance between the tricuspid valve insertions and the RV apex. The strain value at any time point (*t*) with respect to the initial time at RV end-diastole was calculated as $\left(L\left(t\right)-L_0\right)\times100/L_0$, where ${L}_{0}$ is the length of the RV measured between the tracked tricuspid valve insertions and the RV apex at end-diastole; and $L\left(t\right)$, the length at time $t$. As such, the RV fast strain measurement is inherently quantitated in reference to end-diastolic RV length. RV fast global longitudinal strain (GLS)—strain value at end-systole—was obtained from the strain curve; the peak SRs at RV systole (GLSR_s_), early diastole (GLSR_e_), and late diastole (GLSR_a_) were obtained from the SR curve (Fig. 1), which was the first time-derivative of the strain curve obtained by central difference method. Strain and SR parameters from the RV septal and free wall of the 4-chamber view were averaged to obtain mean results for analysis. All strain and SR data were presented as absolute values.

### Conventional feature tracking derived strain

CMR FT was performed by one reader (X.D.Z.) blinded to all participant characteristics and other CMR measurements in deriving RV GLS and global longitudinal SR (GLSR), and global circumferential strain (GCS) using dedicated QStrain software (Version 2.0, Medis BV, Leiden, The Netherlands). RV GLS and GLSR were obtained by endocardial tracking of cine 4-chamber CMR images. RV GCS was derived from the mid-RV short-axis view, which was localized as the level showing the LV short-axis view with both papillary muscles in cross section [4]. The tracking of the RV endocardium was manually corrected in case of inadequate tracking, which is defined by deviation of the contour from the endocardial border on visual assessment by experts [16].

### Cardiopulmonary exercise testing

CPET was performed on a Hewlett–Packard Cosmos treadmill machine as described previously [17]. One metabolic equivalent (MET) was defined as the amount of oxygen consumed while sitting at rest (i.e. 3.5 ml of O_2_ per kilogram body weight per minute). The primary clinical outcome from CPET was peak oxygen consumption (PVO_2_) expressed as an absolute value (ml/kg/min) and as a percentage of predicted PVO_2_ (%predicted PVO_2_). All CPETs are symptom-limited, with PVO_2_ measured by averaging data during the final 15 s of exercise. Normal exercise capacity was defined as > 84 % of predicted PVO_2_ [18].

### Statistical analysis

Statistical analysis was conducted using SPSS (version 17.0, Statistical Package for the Social Sciences, International Business Machines, Inc., Armonk, NY, USA). Continuous data were summarized as mean ± standard deviation (SD) or median (interquartile range (IQR)) and categorical data as frequencies (percentages). Data were compared among the patient and the control groups using an ANOVA *F*-test, followed up with Tukey’s honestly significant difference or Games Howell post hoc test in the event of an overall statistically significant difference in group means. Comparisons between two groups were performed using independent *t* tests for normally distributed data, Mann–Whitney *U* tests for non-normally distributed data, and chi-square test or Fisher’s exact test for categorical data.

Pearson’s *r* correlation, Passing–Bablok non-parametric regression, and Bland–Altman plots were used to assess the agreement of RV fast GLS data with respect to RV GLS. Receiver operator characteristic (ROC) analysis was conducted to assess the clinical discriminative utility of RV strain measurements, and the area under the ROC curve (AUC) was used as a measure of overall discriminative capability. Statistical significance was set at *P* < 0.05.

Intra- and inter-observer variability were studied on a randomly selected subgroup of 30 cases (10 controls, 10 rTOF and 10 PAH) using Bland–Altman analysis and coefficient of variation (CV). The time required for assessment of RV fast GLS and GLS was recorded in the selected 30 cases.

## Results

### Demographics and clinical characteristics of study population

Patients with rTOF were younger than patients with PAH. The female-to-male ratio was 3.7:1 in the PAH patient group. Both rTOF and PAH patients had higher RVEDV index (RVEDVI), RVESV index (RVESVI), and lower RVEF and RV GLS compared to healthy controls. RV GCS was significantly higher in rTOF than in healthy controls. Within the patient groups, RVEF and RVEDVI were higher in rTOF than in PAH, while RVESVI was comparable between rTOF and PAH (Table 1).

**Table 1:** Demographic, clinical characteristics and fast RV strain data of study subjects

Variables	Healthy Controls (n = 75)	rTOF (n = 75)	PAH (n = 75)
Age, years	37 ± 15	31 ± 12*	37 ± 15^#^
Gender, Male/Female	16/59	39/36*	16/59^#^
Body surface area, m^2^	1.6 ± 0.2	1.7 ± 0.2	1.5 ± 0.2*^#^
Diastolic blood pressure, mmHg	74 ± 11	71 ± 9	71 ± 12
Systolic blood pressure, mmHg	126 ± 18	121 ± 16	112 ± 19*^#^
Heart rate, bpm	76 ± 13	78 ± 15	85 ± 16*^#^
WHO class (> 1), n (%)	0 (0 %)	4 (5.3 %)	69 (92 %)*^#^
CMR: left ventricle			
LVEDVI, ml/m^2^	70 ± 11	73 ± 17	72 ± 30
LVESVI, ml/m^2^	26 ± 7	30 ± 11*	32 ± 17*
LVSVI, ml/m^2^	44 ± 6	43 ± 9	41 ± 16
LVEF, %	64 ± 6	59 ± 8*	57 ± 10*
LV mass index, g/m^2^	40 ± 10	48 ± 12*	54 ± 19*^#^
CMR: right ventricle			
RVEDVI, ml/m^2^	73 ± 13	142 ± 30*	122 ± 59*^#^
RVESVI, ml/m^2^	30 ± 10	77 ± 19*	74 ± 46*
RVSVI, ml/m^2^	42 ± 7	65 ± 16*	48 ± 23^#^
RVEF, %	60 ± 7	46 ± 6*	42 ± 14*^#^
RV GCS, %	12.0 ± 4.2	15.4 ± 5.2*	11.0 ± 5.3^#^
RV GLS, %	29.6 ± 3.1	23.8 ± 3.0*	20.4 ± 5.9*^#^
RV GLSR_s_, 1/s	1.5 ± 0.3	1.1 ± 0.2*	1.1 ± 0.4*
RV GLSR_e_, 1/s	1.5 ± 0.4	1.4 ± 0.4	0.8 ± 0.4*^#^
RV GLSR_a_, 1/s	1.0 ± 0.4	0.6 ± 0.3*	1.0 ± 0.5^#^
RV fast GLS, %	24.4 ± 3.0	19.3 ± 2.4*	15.1 ± 4.9*^#^
RV fast GLSR_s_, 1/s	1.2 ± 0.3	0.9 ± 0.2*	0.9 ± 0.3*^#^
RV fast GLSR_e_, 1/s	1.7 ± 0.4	1.4 ± 0.4*	0.8 ± 0.4*^#^
RV fast GLSR_a_, 1/s	1.0 ± 0.3	0.6 ± 0.2*	0.8 ± 0.4*^#^

Data are presented as mean ± SD or n (%)

*CMR* cardiovascular magnetic resonance, *EDVI* end-diastolic volume index, *EF* ejection fraction, *ESVI* end-systolic volume index, *GLS* global longitudinal strain, *GLSR*_s_ peak global longitudinal strain rate during systole, *GLSR*_e_ peak global longitudinal strain rate during early diastole, *GLSR*_a_ peak global longitudinal strain rate during late diastole, *LV* left ventricular, *PAH* pulmonary arterial hypertension, *rTOF* repaired tetralogy of Fallot, *RV* right ventricular, *SVI* stroke volume index, *WHO* World Health Organization

*Significant difference compared to healthy controls; ^#^Significant difference compared to rTOF

In rTOF, the median age at which surgical repair was performed was 4 (IQR 2.2-8.0) years and the median time from surgical repair was 24 (IQR 20.0-31.3) years. 43 patients had undergone transannular patch repair. Patients with subnormal exercise capacity had lower RVEDVI, RVESVI and RV SV index (SVI) (Table 2). LV ejection fraction (LVEF) was comparable between patients with subnormal and normal exercise capacity (59 ± 8 vs. 59 ± 7%, *P* = 0.997).

**Table 2:** Demographic and clinical characteristics in rTOF with normal and subnormal exercise capacity

Variables	Normal exercise capacity (n = 22)	Subnormal exercise capacity (n = 53)	*P* value
Age, years	29 ± 9	32 ± 13	0.243
Gender, Male/Female	12/10	27/26	0.805
Body surface area, m^2^	1.8 ± 0.2	1.6 ± 0.2	0.012
Diastolic blood pressure, mmHg	71 ± 11	71 ± 9	0.809
Systolic blood pressure, mmHg	120 ± 14	121 ± 17	0.719
Heart rate, bpm	78 ± 12	78 ± 16	0.964
Cardiopulmonary exercise testing			
METs	8.3 ± 1.6	6.3 ± 1.5	< 0.001
PVO_2_, ml/kg/min	29.0 ± 5.7	22.1 ± 5.3	< 0.001
% predicted PVO_2_	96.7 ± 10.2	69.9 ± 10.5	< 0.001
CMR: right ventricle			
RVEDVI, ml/m^2^	158 ± 24	135 ± 30	0.002
RVESVI, ml/m^2^	87 ± 14	73 ± 19	0.002
RVSVI, ml/m^2^	72 ± 15	62 ± 16	0.022
RVEF, %	45 ± 5	46 ± 6	0.512

Data are presented as mean ± SD

* CMR* cardiovascular magnetic resonance, *EDVI* end-diastolic volume index, *EF* ejection fraction, *ESVI* end-systolic volume index, *METs* metabolic equivalents, *PVO*_2_ peak oxygen consumption, *RV* right ventricular, *SVI* stroke volume index

Among patients with PAH, those with RV hemodynamic decompensation (PAH-D, n = 20) had higher diastolic blood pressure, lower 6MWD and LVEF compared to those with RV hemodynamic compensation (PAH-C, n = 55) (Table 3). LVEF was significantly lower in PAH-D patients than in those with PAH-C (53 ± 10 vs. 59 ± 10 %, *P* = 0.021). PAH patients with high risk of clinical worsening had lower 6MWD, cardiac index, and RVEF compared to those with low risk (Table 4).

**Table 3:** Demographic and clinical characteristics in PAH with (PAH-C) and without (PAH-D) hemodynamic compensation

Variables	PAH-C (n = 55)	PAH-D (n = 20)	*P* value
Age, years	36 ± 15	41 ± 14	0.246
Gender, Male/Female	9/46	7/13	0.081
Body surface area, m^2^	1.5 ± 0.2	1.6 ± 0.3	0.074
Diastolic blood pressure, mmHg	69 ± 11	76 ± 15	0.026
Systolic blood pressure, mmHg	109 ± 17	118 ± 24	0.074
Heart rate, bpm	85 ± 16	87 ± 16	0.671
WHO class (> 1), n (%)	50 (91 %)	19 (95 %)	0.635
6MWD, m	433 ± 86	380 ± 107	0.049
Right heart catheterization			
RA pressure, mmHg	5 ± 2	16 ± 6	< 0.001
mPAP, mmHg	53 ± 22	62 ± 22	0.137
PCWP, mmHg	9 ± 4	13 ± 4	< 0.001
PVR, Wood units	11 ± 8	15 ± 11	0.146
CMR: right ventricle			
RVEDVI, ml/m^2^	120 ± 56	129 ± 68	0.584
RVESVI, ml/m^2^	72 ± 44	81 ± 50	0.444
RVSVI, ml/m^2^	48 ± 23	48 ± 26	0.910
RVEF, %	43 ± 13	40 ± 15	0.339

Data are presented as mean ± SD or n (%)

*6MWD* six-minute walking distance, *CMR* cardiovascular magnetic resonance, *EDVI* end-diastolic volume index, *EF* ejection fraction, *ESVI* end-systolic volume index, *mPAP* mean pulmonary artery pressure, *PAH-C* compensated pulmonary arterial hypertension, *PAH-D* decompensated pulmonary arterial hypertension, *PCWP* pulmonary capillary wedge pressure, *PVR* pulmonary vascular resistance, *RA* right atrial, *RV* right ventricular, *SVI* stroke volume index, *WHO* World Health Organization

**Table 4:** Clinical characteristics in PAH with low, intermediate and high risks

Variables	Low risk (n = 28)	Intermediate risk (n = 34)	High risk (n = 13)
WHO class (> 1), n (%)	25 (89 %)	31 (91 %)	13 (100 %)
6MWD, m	461 ± 68	403 ± 97*	344 ± 103*
NT-proBNP, pg/ml	140 (60, 308)	605 (223, 1820)*	3475 (1678, 7344)*^#^
Maximal RA area, cm^2^	17 ± 6	24 ± 7*	42 ± 17*^#^
RA pressure, mmHg	5.8 ± 2.8	6.1 ± 3.6	16.8 ± 8.4*^#^
Cardiac index, l/min/m^2^	3.6 ± 2.7	2.5 ± 1.0*	2.1 ± 0.6*
RVEF, %	50 ± 10	39 ± 13*	32 ± 11*

Data are presented as mean ± SD, median (IQR) or n (%)

*6MWD* six-minute walking distance, *EF* ejection fraction, *NT-proBNP* N-terminal pro-brain natriuretic peptide, *RA* right atrial, *RV* right ventricular, *WHO* World Health Organization

*Significant difference compared to low risk; ^#^significant difference compared to intermediate risk

### Feasibility and validation of RV fast GLS

**Fig. 2:**
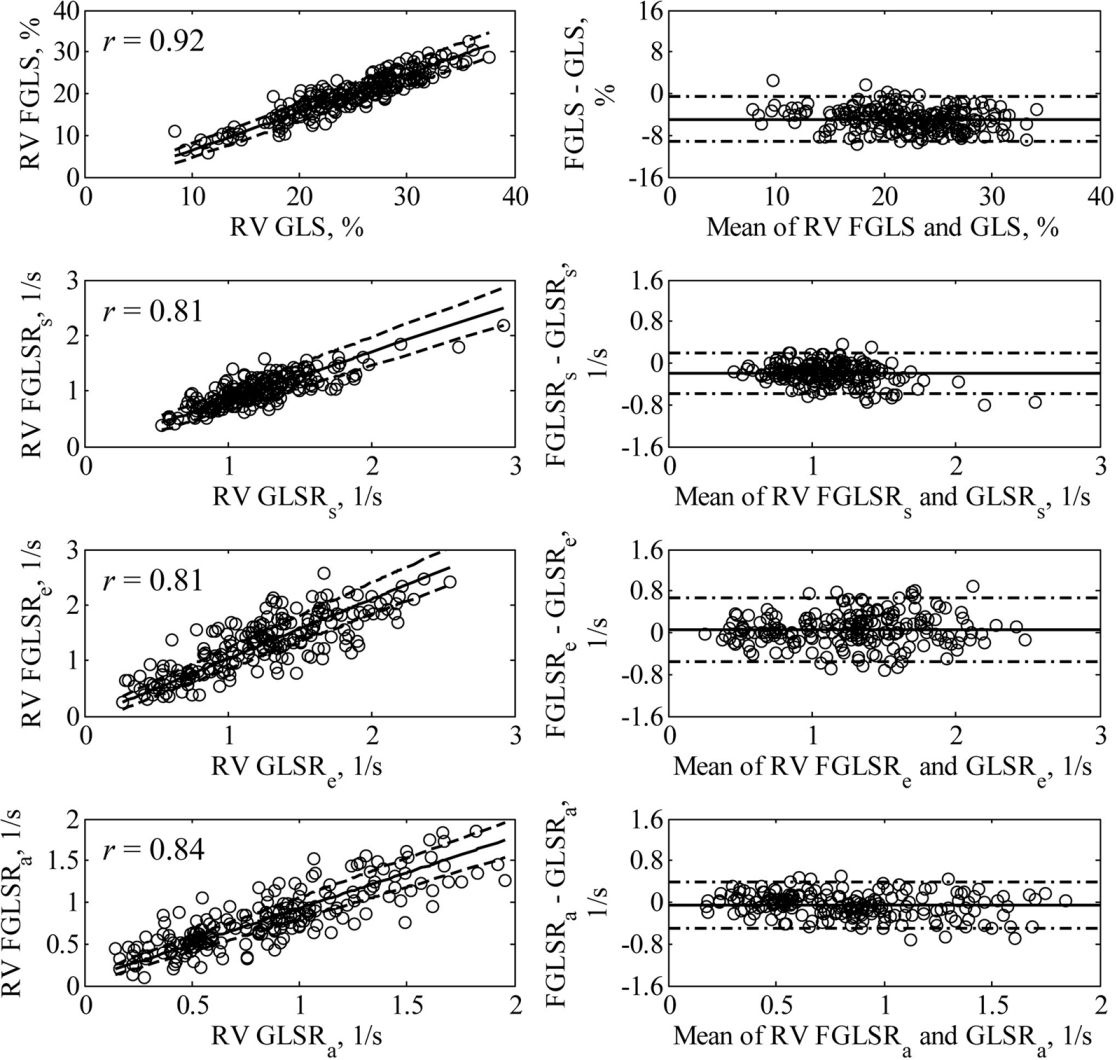
Correlation and Bland-Altman plots (1st row) between RV fast GLS and RV GLS, (2nd row) between RV fast GLSR_s_ and RV GLSR_s_, (3rd row) between RV fast GLSR_e_ and RV GLSR_e_, and (4th row) between RV fast GLSR_a_ and RV GLSR_a_. In correlation plots, solid line and dash lines denote Passing-Bablok non-parametric regression line and 95 % confidence interval, respectively

Fast RV strain analysis was successfully performed in all subjects. The target feature was not correctly located automatically in less than 3 % of total discrete points tracked due to temporal blurring, which necessitated manual correction. RV fast GLS showed overall good correlation with RV GLS (*r* = 0.92, *P* < 0.001) in the entire subject cohort with a bias of − 4.9 % (fast GLS < GLS), and error limits (2SD) of ± 4.3 % (Fig. 2).

Image quality of RV was adequate in all included images with no difference between 1.5 and 3 T. The intra- and inter-observer reproducibility among 10 each of randomly selected 1.5 T and 3 T studies were similar (Table 5). The fast RV strain assessment method was insensitive to small variations in the initial position of RV apex (Pearson correlation: 0.97–0.99; intra-class correlation coefficients: 0.983–0.996; Details are available in Additional file 1).

**Table 5:** Intra- and inter-observer reproducibility for fast global longitudinal strain and strain rate measurements at different field strengths

Variables	Intra-observer CV, %		Inter-observer CV, %	
	1.5 T (n = 10)	3 T (n = 10)	1.5 T (n = 10)	3 T (n = 10)
RV fast GLS, %	3.1	2.9	4.2	3.7
RV fast GLSR_s_, 1/s	6.6	6.1	7.2	7.3
RV fast GLSR_e_, 1/s	6.5	6.8	8.6	8.4
RV fast GLSR_a_, 1/s	7.9	7.2	9.5	10.1

*CV* coefficient of variation, *GLS* global longitudinal strain, *GLSR*_s_ peak global longitudinal strain rate during systole, *GLSR*_e_ peak global longitudinal strain rate during early diastole, *GLSR*_a_ peak global longitudinal strain rate during late diastole, *RV* right ventricular

### RV fast GLS in rTOF and PAH

**Fig. 3:**
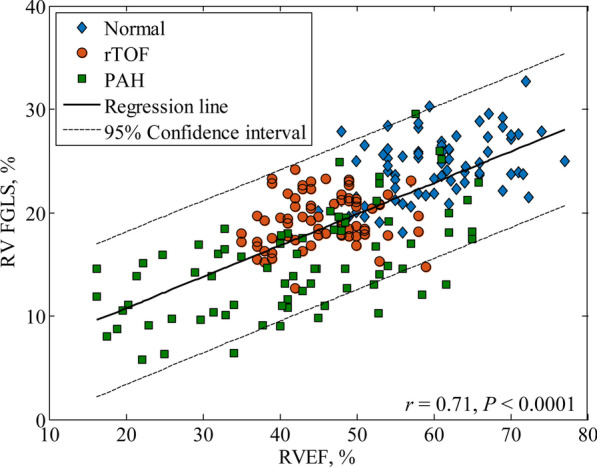
Correlation between right ventricular (RV) fast global longitudinal strain (FGLS) and RV ejection fraction (RVEF) (*r* = 0.71) in the entire subject cohort

A consistent pattern of PAH < rTOF < healthy controls was observed in RV fast GLS and fast GLSR_s_ with significant differences between any two groups (Table 1). There was a modest correlation between RV fast GLS and RVEF in the entire subject cohort (*r* = 0.71, *P* < 0.001) (Fig. 3). The RV fast GLS cut-off values for discriminating various threshold levels of RVEF impairment (< 35%, < 40 % and < 45%) are listed in Additional file 2: Table S1. The AUC was 0.95 when RV fast GLS was used as discriminator of RVEF < 35%.

In both rTOF and PAH patient groups, RV fast GLSR_e_ and fast GLSR_a_ were significantly lower than in the healthy control group. In addition, the RV fast GLSR_e_ was lower in PAH patients than those with rTOF, while fast GLSR_a_ was significantly higher in PAH than in rTOF (Table 1).

### Exercise capacity and its relation to RV fast GLS in rTOF

**Fig. 4:**
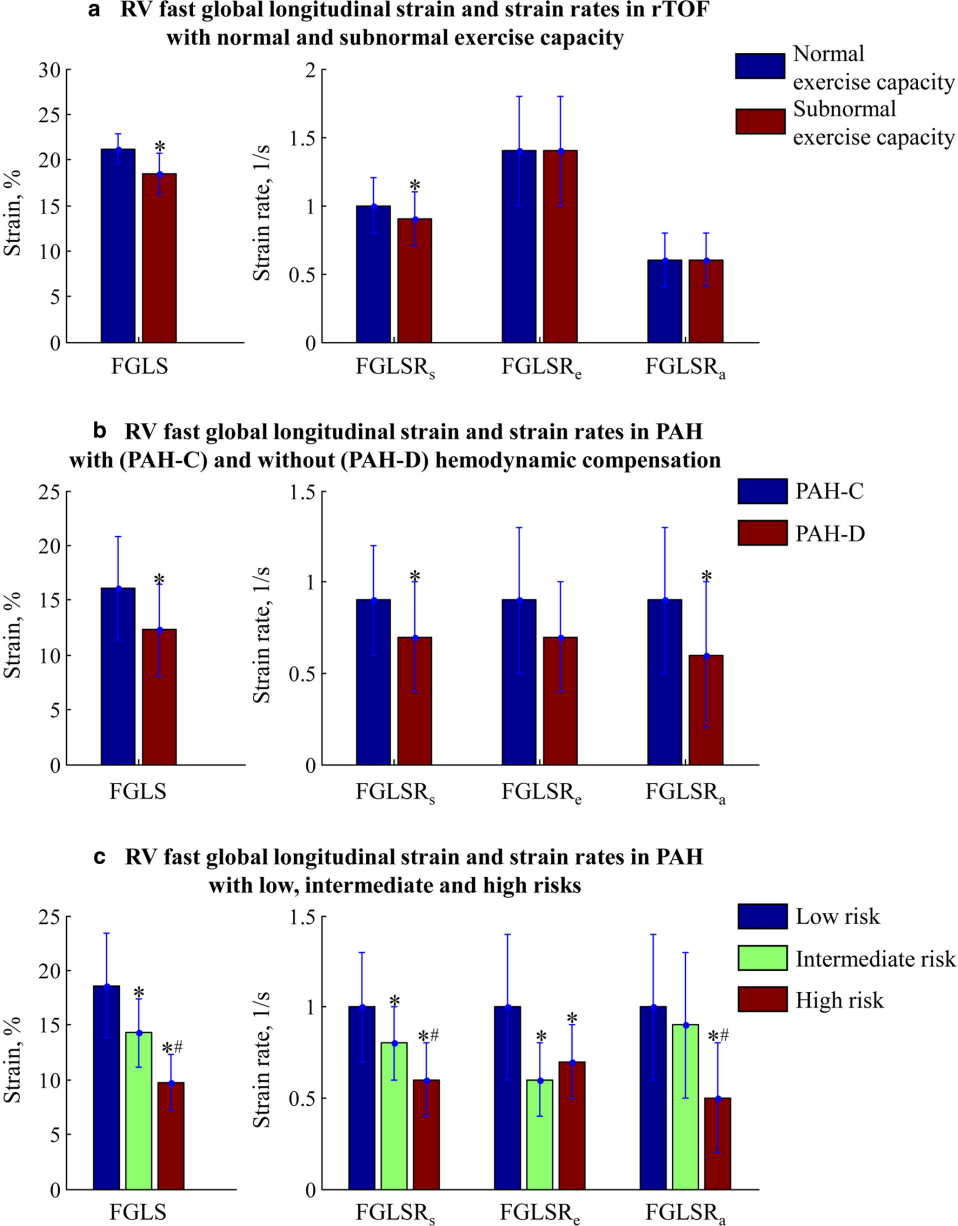
Right ventricular (RV) fast global longitudinal strain (FGLS) and strain rates (FGLSR_s_, FGLSR_e_, and FGLSR_a_) in **a** rTOF patients with normal and subnormal exercise capacity, **b** compensated pulmonary arterial hypertension (PAH-C) and decompensated pulmonary arterial hypertension (PAH-D), **c** PAH with low, intermediate and high risks of clinical worsening. *Significant difference compared to those with normal exercise capacity/PAH-C/low risk; ^#^Significant difference compared to those with intermediate risk

**Fig. 5:**
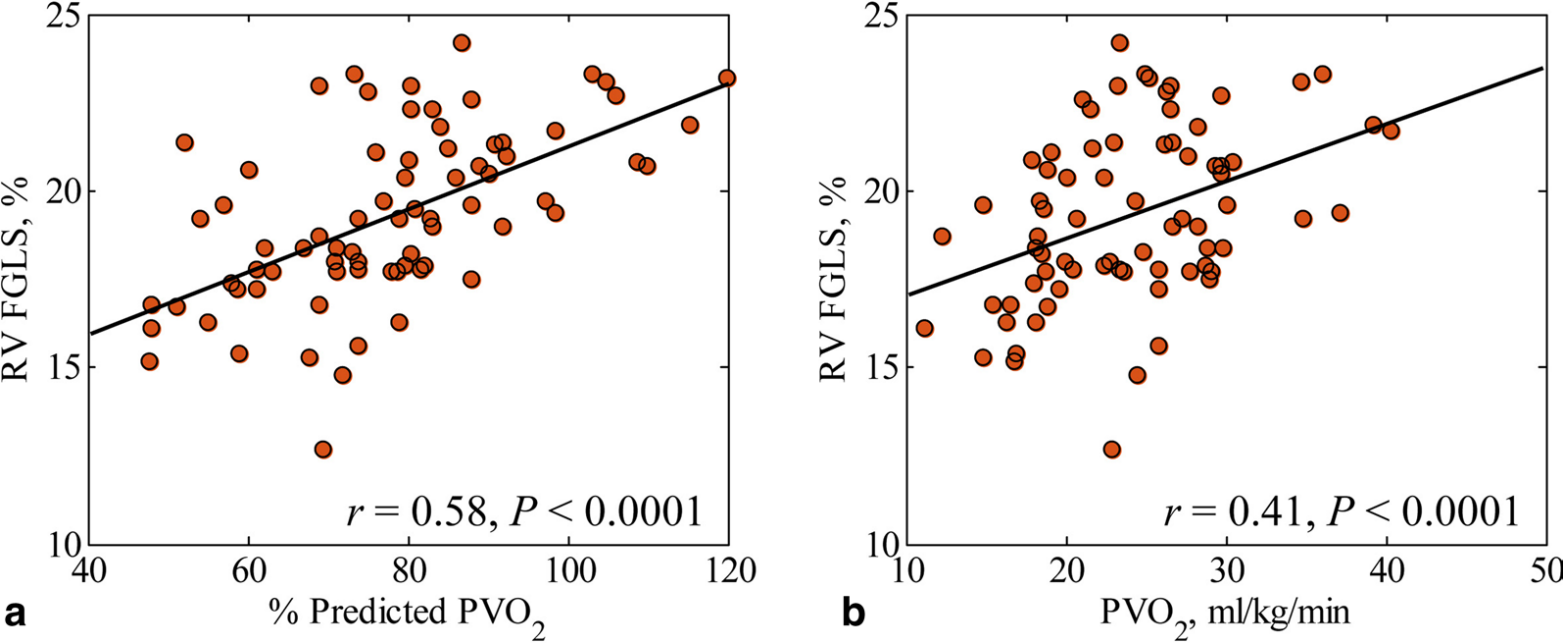
Linear relationship of right ventricular (RV) fast global longitudinal strain (FGLS) to **a** Percent of predicted peak oxygen consumption (% Predicted PVO_2_) (*r* = 0.58) and **b** peak oxygen consumption (PVO_2_) (*r* = 0.41) in rTOF patients

**Fig. 6:**
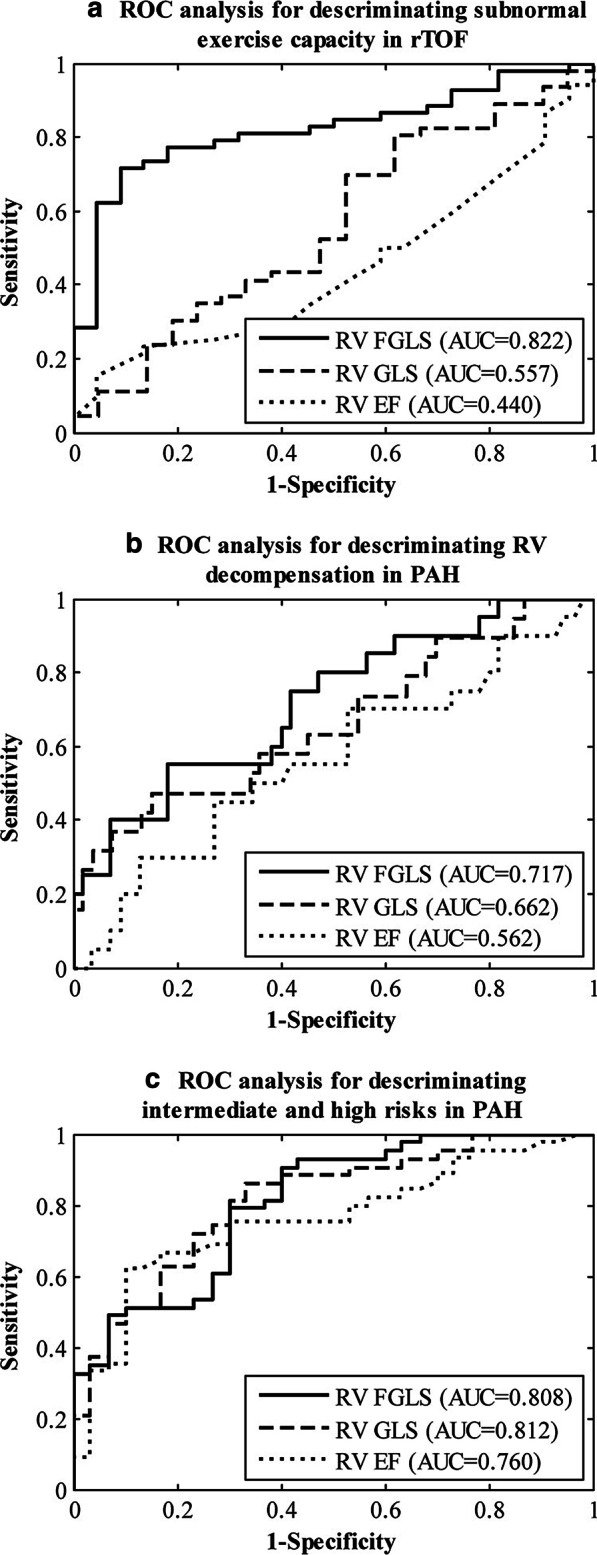
Utility of right ventricular (RV) fast global longitudinal strain (FGLS), conventional RV GLS and RV ejection fraction (RVEF) to discriminate **a** subnormal exercise capacity in rTOF patients, **b** RV decompensation in PAH patients, **c** intermediate and high risks of clinical worsening in PAH patients

On fast RV strain analysis, patients with subnormal exercise capacity had lower RV fast GLS (18.5 ± 2.3 vs. 21.2 ± 1.6%, *P* < 0.001) and fast GLSR_s_ (0.9 ± 0.2 vs. 1.0 ± 0.2 1/s, *P* = 0.004) compared to those with normal exercise capacity (Fig. 4a). Greater RV fast GLS was significantly associated with higher METs (*r* = 0.40, *P* < 0.001), PVO_2_ (*r* = 0.41, *P* < 0.001), and %predicted PVO_2_ (*r* = 0.58, *P* < 0.001) (Fig. 5). ROC analysis demonstrated that RV fast GLS (AUC = 0.822, sensitivity = 72%, specificity = 91%, cut-off = 19.3 %) had better discrimination for subnormal exercise capacity (≤ 84% of predicted PVO_2_) than RV GLS (AUC = 0.557) and RVEF (AUC = 0.440) (Fig. 6a).

### Association of RV fast GLS with hemodynamics, RV decompensation and risk in PAH

RV fast GLS was inversely correlated with invasive RA pressure (*r * = − 0.40, *P *< 0.001), PVR (*r * = − 0.31, *P * = 0.009) and mPAP (*r * = − 0.23, *P* = 0.048) in PAH. In a sub-group analysis, PAH-D had significantly reduced RV fast GLS (12.3 ± 4.2 vs. 16.1 ± 4.7 %, *P* = 0.002), fast GLSR_s_ (0.7 ± 0.3 vs. 0.9 ± 0.3 1/s, *P* = 0.023) and fast GLSR_a_ (0.6 ± 0.4 vs. 0.9 ± 0.4 1/s, *P*=0.004) in comparison to PAH-C (Fig. 4b). ROC analysis demonstrated significantly better performance of RV fast GLS (AUC = 0.717, sensitivity = 75%, specificity = 58%, cut-off = 14.6%) compared with RV GLS (AUC = 0.662) and RVEF (AUC = 0.562) for discriminating PAH-D from PAH-C (Fig. 6b). Reduced fast RV strain and SR measurements were significantly associated with higher risk of clinical worsening in PAH (Fig. 4c). ROC analysis found that RV fast GLS (AUC = 0.808, sensitivity = 79%, specificity = 70%, cut-off = 16.0%) was comparable to RV GLS (AUC = 0.812), but performed better than RVEF (AUC = 0.760) for discriminating patients with intermediate and high risks from those with low risk of clinical worsening (Fig. 6c).

### Reproducibility and time requirement

The intra- and inter-observer strain and SRs derived by the fast method had lower CVs compared to those derived by CMR FT (Table 6). Average assessment time per subject was 32 ± 8 s for the fast method which was significantly shorter than that for CMR FT (75 ± 12 s, *P* < 0.001).

**Table 6:** Intra- and inter-observer reproducibility for RV strain and strain rate measurements derived by fast and conventional feature tracking approaches

Method	RV strain /strain rate	Intra-observer (n = 30)		Inter-observer (n = 30)	
		Bias (limits of agreement)	CV (%)	Bias (limits of agreement)	CV (%)
Fast strain approach	Fast GLS, %	− 0.1 (− 1.6, 1.4)	2.9	− 0.3 (− 2.3, 1.8)	3.9
	Fast GLSR_s_, 1/s	− 0.04 (− 0.20, 0.12)	6.3	− 0.03 (− 0.22, 0.17)	7.0
	Fast GLSR_e_, 1/s	− 0.02 (− 0.23, 0.20)	6.5	− 0.03 (− 0.30, 0.23)	8.1
	Fast GLSR_a_, 1/s	− 0.03 (− 0.21, 0.15)	7.5	− 0.03 (− 0.27, 0.20)	9.6
Feature tracking approach	GLS, %	− 0.2 (− 4.3, 3.9)	6.0	− 0.1 (− 5.3, 5.1)	7.5
	GLSR_s_, 1/s	− 0.02 (− 0.33, 0.29)	9.3	− 0.02 (− 0.36, 0.32)	10.1
	GLSR_e_, 1/s	0.10 (− 0.23, 0.43)	12.1	0.04 (− 0.40, 0.47)	13.3
	GLSR_a_, 1/s	− 0.06 (− 0.37, 0.25)	12.9	− 0.04 (− 0.40, 0.32)	14.4

*CV* coefficient of variation, *GLS* global longitudinal strain, *GLSR*_s_ peak global longitudinal strain rate during systole, *GLSR*_e_ peak global longitudinal strain rate during early diastole, *GLSR*_a_ peak global longitudinal strain rate during late diastole, *RV* right ventricular

## Discussion

The current study demonstrated that RV longitudinal strain can be rapidly quantified by tracking 3 distinct anatomical points in standard CMR sequences with greater reproducibility, shorter analysis time, and comparable diagnostic and prognostic capabilities compared to conventional CMR FT. Greater RV fast GLS was found to correlate significantly with higher METs, PVO_2_, and %predicted PVO_2_ in rTOF patients. Among PAH patients, RV fast GLS and fast GLSR were significantly reduced in patients with hemodynamically decompensated RV compared to those with compensated RV. Lower RV fast GLS and fast GLSR measurements were significantly associated with higher risk of clinical worsening in PAH patients.

### RV function and exercise capacity in rTOF

RV dilation alone reflects little, but RV function relates to exercise capacity and functional health status. The current study applied the fast strain assessment by CMR to directly measure RV longitudinal shortening, and showed significant association between RV fast GLS and exercise performance in rTOF patients. The traditional RV GLS was also shown to be related to %predicted PVO_2_ (*r* = 0.26, *P* = 0.033). Our finding agreed with an earlier study by Kempny et al. [19] who showed that CMR based RV GLS was significantly related to exercise capacity. Another study found that RV GLS by speckle tracking echocardiography (STE) correlated moderately well (*r* = 0.42, *P* < 0.01) with exercise capacity, and was an important predictor of clinical outcome in patients with congenital heart disease [20]. Similar to observations in adults, pediatric patients with rTOF exhibited reduced STE-derived RV systolic strain in association with decreased %predicted PVO_2_ (*r* = 0.66, *P* = 0.0001) [21].

Interestingly, we found circumferential RV strain to be higher in rTOF patients compared with healthy controls. Previous research dichotomized the contribution to RV SV as 80% being generated by RV longitudinal function and the remaining 20% by “radial” function [22]. The latter in contemporary understanding probably comprises a combination of non-longitudinal components like circumferential and radial motion. The current study found significantly decreased RV GLS in rTOF patients and increased GCS that more than compensate to maintain the significantly larger RV SV in rTOF. The association between RV GCS and exercise capacity in rTOF patients warrants further investigation.

### RV function in PAH

The clinical gold standard for assessing disease severity in the pressure-overloaded state remains invasive hemodynamics [23]. PAH-D is characterized by high RA pressure in addition to high mPAP. We demonstrated a strong association between RV fast GLS and PAH decompensation as well as the risk of clinical worsening. RV fast GLS was lower in PAH-D compared to PAH-C and correlated with invasive hemodynamic measurements. This is consistent with a previous study [12] that demonstrated significant stepwise decrement in the peak RV free wall longitudinal strain as measured by tissue Doppler imaging among controls, and PAH patients with compensated and decompensated RV function. Our findings that RV fast GLS differentiated PAH from controls as well as PAH-D from PAH-C with good sensitivity and specificity suggest that RV fast GLS can be a useful non-invasive parameter for identifying hemodynamically significant PAH and for monitoring RV function in such patients. The attenuation of RV fast GLS—an important measure of RV longitudinal contractility—may point towards risks of RV failure in the setting of chronic pressure overload.

Recent guidelines recommend a multidimensional approach in comprehensive risk assessment for PAH. Patients classified as intermediate and high risk have higher estimated 1-year mortality [11]. We found lower fast RV strain and SRs to be significantly associated with higher risks, suggesting that fast RV strain measurements may provide diagnostic capability to discriminate the risk level of PAH patients and therefore potentially add incremental value for the serial monitoring of RV function with disease progression or after PAH-specific drug intervention. The definitive proof awaits further investigation.

### Advantages of RV fast GLS over RVEF and RV GLS

Assessment of RV contractile function is of great clinical importance, particularly in the monitoring and management of patients with congenital heart disease. Volume calculation and estimation of RVEF may be relatively insensitive for detecting changes in RV systolic function with disease [24]. In patients with RV volume or pressure overloading, the complex RV geometry can mask the presence of reduced deformation, resulting in an apparently preserved or less impaired RVEF. Hence, strain may be a more accurate marker of systolic function in these patients.

Typical RV CMR FT algorithm ignores the septum and tracks the RV free wall during a cardiac cycle, resulting in free wall longitudinal strain. In the present study, we performed RV fast GLS and traditional RV GLS measurements by tracking both RV free wall and interventricular septum. The accuracy of traditional RV GLS is degraded in subjects with vigorous tricuspid annular motion, as contour tracking of the RV free wall segment adjacent to the tricuspid valve in long-axis view is adversely affected [5]. The RV fast GLS presented in this study was less dependent on RV geometry than were endocardial measurements such as RV EF and RV GLS. The result data showed that our fast approach is not only faster but also more reproducible compared to conventional CMR FT.

### Limitations

RV geometry is complex and it is virtually impossible to capture the entirety of 3D RV strain using a single parameter. RV fast GLS is an index of global RV function in the longitudinal direction and does not provide information on circumferential and radial strains such as those integrated in area strain [25]. However, RV shortening is predominantly longitudinal. In comparison to RV GLS, consistently lower values for RV fast GLS were reflected in the Bland-Altman plot (bias − 4.9 %, fast GLS < GLS), possibly attributable to a geometric factor [10]. Due to the double trapezoid conformation of the RV, the true apex of the RV is hard to pinpoint. Here, we adopted the expedient convention where its location is defined by proximity to the LV apex, which is close to but separated from the RV apex by the interventricular septal wall on the standard cine 4-chamber view [26]. In the present study, CPET was performed only in the rTOF patient group.

## Conclusions

RV fast GLS agrees with conventional RV CMR FT strain and appears to be more reproducible. The RV fast strain and SR parameters identified abnormalities of RV function in rTOF and PAH and were associated with exercise capacity, RV decompensation and clinical risks in these patients.

## Supplementary Information

**Additional file 1.** Fast global longitudinal strain: details about the anatomical point tracking and the methodological considerations and robustness analysis on RV apical point selection

**Additional file 2.** RV FGLS cut-off values with AUC, sensitivity and specificity for discriminating RVEF impairment < 35 %, < 40 % and < 45 %

## Data Availability

All data generated or analysed during this study are included in this published article [and its supplementary information files].

## Abbreviations

6MWD: Six-minute walking distance
AUC: Area under ROC curves
CMR: Cardiovascular magnetic resonance
CPET: Cardiopulmonary exercise testing
CV: Coefficient of variation
EDV: End-diastolic volume
EDVI: End-diastolic volume index
EF: Ejection fraction
ESV: End-systolic volume
ESVI: End-systolic volume index
FT: Feature tracking
GCS: Global circumferential strain
GLS: Global longitudinal strain
GLSR: Global longitudinal strain rate
GLSRa: Peak global longitudinal strain rate at late diastole
GLSRe: Peak global longitudinal strain at early diastole
GLSRs: Peak global longitudinal strain at right ventricular systole
LV: Left ventricle/left ventricular
LVEF: Left ventricular ejection fraction
MET: Metabolic equivalent
mPAP: Mean pulmonary artery pressure
NYHA: New York Heart Association
PAH: Pulmonary arterial hypertension
PAH-C: Pulmonary arterial hypertension with hemodynamically compensated RV
PAH-D: Pulmonary arterial hypertension with hemodynamically decompensated RV
PVO_2_: Peak oxygen consumption
PVR: Pulmonary vascular resistance
RA: Right atrium/right atrial
ROC: Receiver operating characteristic
rTOF: Repaired tetralogy of Fallot
RV: Right ventricle/right ventricular
RVEF: Right ventricular ejection fraction
SR: Strain rate
STE: Speckle tracking echocardiography
SV: Stroke volume
SVI: Stroke volume index

## References

[1] Sanz J, Sánchez-Quintana D, Bossone E, Bogaard HJ, Naeije R (2019). Anatomy, function, and dysfunction of the right ventricle: JACC state-of-the-art review. J Am Coll Cardiol.

[2] Geva T (2014). Is MRI the preferred method for evaluating right ventricular size and function in patients with congenital heart disease? MRI is the preferred method for evaluating right ventricular size and function in patients with congenital heart disease. Circ Cardiovasc Imaging.

[3] Schuster A, Hor KN, Kowallick JT, Beerbaum P, Kutty S (2016). Cardiovascular magnetic resonance myocardial feature tracking: concepts and clinical applications. Circ Cardiovasc Imaging.

[4] De Siqueira MEM, Pozo E, Fernandes VR, Sengupta PP, Modesto K, Gupta SS (2016). Characterization and clinical significance of right ventricular mechanics in pulmonary hypertension evaluated with cardiovascular magnetic resonance feature tracking. J Cardiovasc Magn Reson.

[5] Bhave NM, Visovatti SH, Kulick B, Kolias TJ, McLaughlin VV (2017). Right atrial strain is predictive of clinical outcomes and invasive hemodynamic data in group 1 pulmonary arterial hypertension. Int J Cardiovasc Imaging.

[6] Bourfiss M, Vigneault DM, Aliyari Ghasebeh M, Murray B, James CA, Tichnell C (2017). Feature tracking CMR reveals abnormal strain in preclinical arrhythmogenic right ventricular dysplasia/cardiomyopathy: a multi software feasibility and clinical implementation study. J Cardiovasc Magn Reson.

[7] Leng S, Ge H, He J, Kong LC, Yang YN, Yan FH (2020). Long-term prognostic value of cardiac MRI left atrial strain in ST-segment elevation myocardial infarction. Radiology.

[8] Leng S, Tan RS, Zhao XD, Allen JC, Koh AS, Zhong L (2018). Validation of a rapid semi-automated method to assess left atrial longitudinal phasic strains on cine cardiovascular magnetic resonance imaging. J Cardiovasc Magn Reson.

[9] Leng S, Dong Y, Wu Y, Zhao XD, Ruan W, Zhang GC (2019). Impaired CMR-derived rapid semi-automated right atrial longitudinal strain is associated with decompensated hemodynamics in pulmonary arterial hypertension. Circ Cardiovasc Imaging.

[10] Leng S, Tan RS, Zhao XD, Allen JC, Koh AS, Zhong L (2020). Fast long-axis strain: a simple, automatic approach for assessing left ventricular longitudinal function with cine cardiovascular magnetic resonance. Eur Radiol.

[11] Galiè N, Humbert M, Vachiery JL, Gibbs S, Lang I, Torbicki A (2016). 2015 ESC/ERS Guidelines for the diagnosis and treatment of pulmonary hypertension: The Joint Task Force for the Diagnosis and Treatment of Pulmonary Hypertension of the European Society of Cardiology (ESC) and the European Respiratory Society (ERS): Endorsed by: Association for European Paediatric and Congenital Cardiology (AEPC), International Society for Heart and Lung Transplantation (ISHLT). Eur Heart J.

[12] Simon MA, Rajagopalan N, Mathier MA, Shroff SG, Pinsky MR, López-Candales A (2009). Tissue Doppler imaging of right ventricular decompensation in pulmonary hypertension. Congest Heart Fail.

[13] Schulz-Menger J, Bluemke DA, Bremerich J, Flamm SD, Fogel MA, Friedrich MG (2020). Standardized image interpretation and post-processing in cardiovascular magnetic resonance – 2020 update. J Cardiovasc Magn Reson.

[14] Leng S, Zhao XD, Huang FQ, Wong JI, Su BY, Allen JC (2015). Automated quantitative assessment of cardiovascular magnetic resonance-derived atrioventricular junction velocities. Am J Physiol Heart Circ Physiol.

[15] Leng S, Jiang M, Zhao XD, Allen JC, Kassab GS, Ouyang RZ (2016). Three-dimensional tricuspid annular motion analysis from cardiac magnetic resonance feature-tracking. Ann Biomed Eng.

[16] Schuster A, Stahnke VC, Unterberg-Buchwald C, Kowallick JT, Lamata P, Steinmetz M (2015). Cardiovascular magnetic resonance feature-tracking assessment of myocardial mechanics: intervendor agreement and considerations regarding reproducibility. Clin Radiol.

[17] Yap J, Tan JL, Le TT, Gao F, Zhong L, Liew R (2014). Assessment of left ventricular preload by cardiac magnetic resonance imaging predicts exercise capacity in adult operated tetralogy of Fallot: a retrospective study. BMC Cardiovasc Disor.

[18] Albouaini K, Egred M, Alahmar A, Wright DJ (2007). Cardiopulmonary exercise testing and its application. Heart.

[19] Kempny A, Fernández-Jiménez R, Orwat S, Schuler P, Bunck AC, Maintz D (2012). Quantification of biventricular myocardial function using cardiac magnetic resonance feature tracking, endocardial border delineation and echocardiographic speckle tracking in patients with repaired tetralogy of Fallot and healthy controls. J Cardiovasc Magn Reson.

[20] Ladouceur M, Redheuil A, Soulat G, Delclaux C, Azizi M, Patel M (2016). Longitudinal strain of systemic right ventricle correlates with exercise capacity in adult with transposition of the great arteries after atrial switch. Int J Cardiol.

[21] Alghamdi MH, Mertens L, Lee W, Yoo SJ, Grosse-Wortmann L (2013). Longitudinal right ventricular function is a better predictor of right ventricular contribution to exercise performance than global or outflow tract ejection fraction in tetralogy of Fallot: a combined echocardiography and magnetic resonance study. Eur Heart J Cardiovasc Imaging.

[22] Carlsson M, Ugander M, Heiberg E, Arheden H (2007). The quantitative relationship between longitudinal and radial function in left, right, and total heart pumping in humans. Am J Physiol Heart Circ Physiol.

[23] Champion HC, Michelakis ED, Hassoun PM (2009). Comprehensive invasive and noninvasive approach to the right ventricle-pulmonary circulation unit state of the art and clinical and research implications. Circulation.

[24] Chen SS, Keegan J, Dowsey AW, Ismail T, Wage R, Li W (2011). Cardiovascular magnetic resonance tagging of the right ventricular free wall for the assessment of long axis myocardial function in congenital heart disease. J Cardiovasc Magn Reson.

[25] Zhong L, Gobeawan L, Su Y, Tan JL, Ghista D, Chua T (2012). Right ventricular regional wall curvedness and area strain in patients with repaired tetralogy of Fallot. Am J Physiol Heart Circ Physiol.

[26] Valsangiacomo Buechel ER, Mertens LL (2012). Imaging the right heart: the use of integrated multimodality imaging. Eur Heart J.

