# Supplementary material for: Cardiovascular magnetic resonance‐assessed fast global longitudinal strain parameters add diagnostic and prognostic insights in right ventricular volume and pressure loading disease conditions
Source: J Cardiovasc Magn Reson. 2021 Apr 1;23:38. doi: 10.1186/s12968-021-00724-5 (PMC8015087; doi:10.1186/s12968-021-00724-5)
Supplement: Supplementary file 1 — Additional file 1. Fast global longitudinal strain: details about the anatomical point tracking and the methodological considerations and robustness analysis on RV apical point selection [file 12968_2021_724_MOESM1_ESM.pdf]

Additional file 1: Fast global longitudinal strain (FGLS)

Fast right ventricular (RV) strain assessment was performed by automatically tracking the distance (L) from the medial and lateral tricuspid valve insertions to the RV epicardial apex on CMR 4-chamber view (Figure 1 of the paper). Briefly, squares (called masks, Figure 1 of the paper) containing the anatomical points of interest (tricuspid valve insertions and RV epicardial apex) were manually drawn in the RV end-diastolic frame (1st frame). These masks were automatically tracked forward in time to the next cardiac frame (2nd frame) using a template matching algorithm [1]. The location of the best match was used to update the masks in the target frame (2nd frame) and the same procedure was automatically executed iteratively for all subsequent frames. This forward tracking provided a first set of tracked point candidates. The initial masks drawn manually in the 1st frame were also tracked backward in time to the previous cardiac frame (the last frame – frame 30 in Figure 1 of the paper) and subsequently in all frames of the cardiac cycle, providing another set of possible point-of-interest localizations. The final trajectory result was calculated by averaging those from both forward and backward tracking.

We follow the convention where the RV apex location is determined by its proximity to the left ventricular (LV) apex. The RV apex is close to but separated from the LV apex by the interventricular septal wall, which can be viewed on the 4-chamber view [2, 3]. In our work, we tracked the RV apex over time. In rTOF, RV dilatation is typically generalized and the RV apex is less likely to become aneurysmal and distorted. Even if that occurs, we would continue to track the RV apex as determined by its spatial relationship to the LV apex on the 4-chamber view. We believe it is more important the method be standardized and reproducible than customizing to individual cases for differences that are likely to be small, if present. Such an

approach is not without precedence. The investigators in a prior study [4] used the distance between the LV apex and tricuspid valve plane to track RV longitudinal motion.

Additional analysis has been performed in a subset of patients and normal controls with ten subjects in each category to test the robustness of the method via slight variations on the initial mask position at RV apex. The robustness test was conducted as follows:

1) The position of original mask at RV apex in the initial frame is denoted as (x_0, y_0). The size of the mask is $w \times h$.
2) The new mask position (x_n, y_n) was obtained by slightly varying the original as $(x_n = x_0 + \Delta w, y_n = y_0 + \Delta h)$, where Δw and Δh were randomly generated between $-0.2w$ and $+0.2w$, and between $-0.2h$ and $+0.2h$, respectively.
3) Automatic tracking was executed with the new mask without manual intervention.
4) Fast long-axis strain and strain rate measurements were then derived and compared with the original results.

Good agreements were observed for all strain and strain rate measurements between the two sets of results with high Pearson correlation values (0.97-0.99) and intra-class correlation coefficients (0.983-0.996), indicating that the fast RV strain assessment method was insensitive to small variations in the initial position of RV apex.

Reference

1. Gonzalez RC, Woods RE (2018) Digital image processing. 4th ed. NY: Pearson/Prentice Hall.
2. Ho SY, Nihoyannopoulos P. Anatomy, echocardiography, and normal right ventricular dimensions. Heart. 2006;92 Suppl 1:i2-13.

3. Valsangiacomo Buechel ER, Mertens LL. Imaging the right heart: the use of integrated multimodality imaging. Eur Heart J. 2012;33:949-60.
4. Arenja N, Riffel JH, Djiokou CN, Andre F, Fritz T, Halder M, et al. Right ventricular long axis strain-validation of a novel parameter in non-ischemic dilated cardiomyopathy using standard cardiac magnetic resonance imaging. Eur J Radiol. 2016;85:1322-8.
