# Supplementary material for: Cardiovascular magnetic resonance‐assessed fast global longitudinal strain parameters add diagnostic and prognostic insights in right ventricular volume and pressure loading disease conditions
Source: J Cardiovasc Magn Reson. 2021 Apr 1;23:38. doi: 10.1186/s12968-021-00724-5 (PMC8015087; doi:10.1186/s12968-021-00724-5)
Supplement: Supplementary file 2 — Additional file 2. RV FGLS cut-off values with AUC, sensitivity and specificity for discriminating RVEF impairment < 35 %, < 40 % and < 45 % [file 12968_2021_724_MOESM2_ESM.docx]

**Additional file 2: Table S1.** RV fast GLS cut-off values with AUC, sensitivity and specificity for discriminating RVEF impairment <35%, <40% and <45%

| **RVEF** | **RV fast GLS, %** | | | |
| --- | --- | --- | --- | --- |
|  | **AUC** | **Sensitivity** | **Specificity** | **Cut-off** |
| <35% | 0.946 | 100 | 82 | 17 |
| <40% | 0.878 | 88 | 83 | 18 |
| <45% | 0.833 | 76 | 74 | 19 |

AUC, area under the receiver operating characteristic curve; GLS, global longitudinal strain; RVEF, right ventricular ejection fraction.
